# An Efficient Bio-Receptor Layer Combined with a Plasmonic Plastic Optical Fiber Probe for Cortisol Detection in Saliva

**DOI:** 10.3390/bios14070351

**Published:** 2024-07-19

**Authors:** Francesco Arcadio, Mimimorena Seggio, Rosalba Pitruzzella, Luigi Zeni, Alessandra Maria Bossi, Nunzio Cennamo

**Affiliations:** 1 Department of Engineering, University of Campania Luigi Vanvitelli, Via Roma 29, 81031 Aversa, Italy; francesco.arcadio@unicampania.it (F.A.); rosalba.pitruzzella@unicampania.it (R.P.); luigi.zeni@unicampania.it (L.Z.); 2 Department of Biotechnology, University of Verona, Strada Le Grazie 15, 37134 Verona, Italy; mimimorena.seggio@univr.it

**Keywords:** cortisol, stress, optical sensors, biosensor, surface plasmon resonance (SPR), plastic optical fibers (POFs), glucocorticoid receptor (GR), point-of-care test (POCT)

## Abstract

Cortisol is a clinically validated stress biomarker that takes part in many physiological and psychological functions related to the body’s response to stress factors. In particular, it has emerged as a pivotal tool for understanding stress levels and overall well-being. Usually, in clinics, cortisol levels are monitored in blood or urine, but significant changes are also registered in sweat and saliva. In this work, a surface plasmon resonance probe based on a D-shaped plastic optical fiber was functionalized with a glucocorticoid receptor exploited as a highly efficient bioreceptor specific to cortisol. The developed plastic optical fiber biosensor was tested for cortisol detection in buffer and artificial saliva. The biosensor response showed very good selectivity towards other hormones and a detection limit of about 59 fM and 96 fM in phosphate saline buffer and artificial saliva, respectively. The obtained detection limit, with a rapid detection time (about 5 min) and a low-cost sensor system, paved the way for determining the cortisol concentration in saliva samples without any extraction process or sample pretreatment via a point-of-care test.

## 1. Introduction

In healthcare and physiological research, the measurement of cortisol levels has emerged as a pivotal tool for understanding stress levels, hormonal regulation, and overall well-being [1,2]. Cortisol is a glucocorticoid steroid hormone secreted by the adrenal gland and regulated by the hypothalamic–pituitary–adrenal system [3]. Following a well-established circadian rhythm, the hormone concentration varies throughout the day: it stays lower at midnight, begins to rise in the early morning, and then decreases once more during the day [4]. In serum, concentration levels between 136 nM and 690 nM in the morning and between 55 nM and 386 nM in the evening have been monitored [5,6]. However, the range of values can change as a function of the biofluid analyzed (i.e., blood, urine, saliva, sweat, etc.) [7]. Moreover, the hormone is a clinically validated stress biomarker that takes part in many physiological and psychological functions associated with the body’s response to stress factors [8], such as gluconeogenesis, lipolysis and proteolysis, and immunosuppression [2,9,10].

Usually, in clinics, cortisol levels are monitored in blood or urine, but significant changes have also been registered in sweat and saliva [11]. The hormone is commonly monitored and quantified via ultraviolet spectroscopy, gas chromatography−mass spectrometry, electrochemical and enzyme-linked immunosorbent assays (ELISA), radioimmunoassays, and surface-enhanced Raman spectroscopy [12]. These methods allow for sensitive cortisol detection in biofluids, but they have a number of disadvantages, including a high sample volume (0.2–2 mL), lengthy analysis times, the necessity for specialized staff, and the need for expensive instrumentation [13]. Moreover, the traditional monitoring methods pose discomfort to individuals and limit the frequency and practicality of measurements, hindering the ability to gain real-time insights into stress patterns. In this context, saliva cortisol monitoring offers the possibility to obtain valuable information about an individual’s stress response and overall health non-invasively and cost-effectively [14].

Among several optical methods for cortisol detection summarized in a recent review [15], surface plasmon resonance (SPR) has gained significant interest due to the notably low limit of detection (LOD) achieved [16,17,18]. In particular, plastic optical fiber (POF) SPR sensors have attracted even more attention due to their small size, portability, lightweight sensing layout, remote sensing, and high sensitivity [19]. Plasmonic POF-based sensors were employed in diverse fields of application, including environmental [20], medical, and biomedical ones [21], and, in particular, hormone monitoring [22]. Recently, a cortisol biosensor based on an SPR-POF probe functionalized with an anti-cortisol antibody was developed [23], obtaining a LOD of 0.2 pg/mL (552 fM), which is lower than those achieved by other plasmonic biosensors already reported in the literature [24,25]. In [23], the plasmonic platform based on modified POFs is the same as that developed in [26], where a photoresist buffer layer is deposited on the exposed core of the D-shaped POF area to improve the optical sensitivity of the SPR D-shaped POF platform.

In this work, in order to further improve the sensor performance in cortisol monitoring, the same SPR-POF platform based on a D-shaped POF [23,26] is functionalized with a different receptor, i.e., a glucocorticoid receptor (GR). Even if the SPR-POF probe denotes an optical sensitivity equivalent to [23], the GR receptor exhibits a higher efficiency with respect to the antibody receptor used in [23]. In particular, the efficiency represents a peculiar aspect of the receptor layer. It denotes how the binding of an analyte at a certain concentration is transformed into a refractive index (RI) variation at the gold surface [27]. In this frame, the GR is a nuclear polypeptide receptor which regulates several functions of glucocorticoids as a ligand-dependent transcription factor. The binding of specific agonists, such as cortisol for the GR, induces specific conformational changes in the protein, leading to GR activation and subsequent regulation of gene transcription [28]. For this feature, the deformable at-binding GR was chosen as the biosensor’s recognition element. In fact, it was expected that the higher deformation of GR at binding with cortisol with respect to the antibody would produce higher variation in terms of RI at the plasmonic gold surface, achieving higher efficiency and, thus, improved performance.

The so-produced biosensor was tested in cortisol monitoring in both phosphate buffer saline (PBS) and simulated saliva, without the need for sample pretreatment procedures, to assess the response in a complex matrix, opening the path to a real application scenario. In this work, the low-cost POF-based plasmonic chip, the setup, and the performances of the bio-receptor layer are exploited to develop and test a point-of-care test (POCT) that is useful for measuring the analyte in saliva.

## 2. Materials and Methods

### 2.1. Chemicals

Recombinant human glucocorticoid receptor (AMab82089) (GR) was purchased from abcam (Cambridge, UK). PBS 10 mM with a pH of 7.4, cortisol, estradiol, progesterone, artificial saliva for pharmaceutical research, α-lipoic acid, N-(3-dimethylaminopropyl)-N′-ethylcarbodiimide hydrochloride (EDC), N-hydroxysuccinimide (NHS), ethanolamine, and 2-(N-morpholino)ethanesulfonic acid (MES) were bought from Merck-Sigma (Darmstadt, Germany). 

### 2.2. SPR-POF Probe

The SPR D-shaped POF platform was produced according to Cennamo et al. [23,26]. Specifically, a 1 mm POF was glued into a resin support and lapped by two polishing sheets with different grits (5 µm and 1 µm) to obtain an area of the POF with the shape of a “D”. Onto this, it was then deposited by spin-coating a layer with a high refractive index (Microposit S1813, Chimie Tech Services, Antony, France) to enhance the plasmonic performance [26]. As a final step, the surface was then covered by a thin gold metal film with a thickness of around 60 nm. This process was carried out using a sputter coater (Safematic CCU-010, Zizers, Switzerland). This plasmonic POF-based probe was the same one used in [23]. This was intended to compare the biosensors’ performances in cortisol detection when using the same SPR-POF probe coupled with different bioreceptor layers.

### 2.3. GR Functionalization Protocol of SPR-POF Chip

The coupling between the GR and the plasmonic probe was carried out by following the procedure described by Arcadio et al. [22].

After a rinsing phase with MilliQ water, the gold SPR-POF surface was treated overnight at room temperature (r.t.) with α-lipoic acid at a concentration of 0.3 mM in 8% ethanolic solution, producing a self-assembled monolayer (SAM). In the second step, the so-produced surface was activated with EDC/NHS at a concentration of 10 mM in MES buffer (50 mM, pH 5.5) for twenty minutes at r.t. Then, GR (15 µL, 0.88 mg/mL) were incubated for 2 h on the activated surface in a confined chamber. Lastly, the unreacted sites were blocked by incubating the surface with ethanolamine at a concentration of 1 mM in water for thirty minutes at r.t. At the end of the process, the sensor was rinsed with PBS and kept in PBS at a controlled temperature of 4 °C. The functionalization process of the GR-SPR-POF platform was evaluated by monitoring the variations in the plasmonic wavelength, which were computed in relation to the non-functionalized chip, and considering PBS as the surrounding solution.

The error bars were calculated as the maximum value of the experimentally measured standard deviation, which was attained by testing three biosensors in similar conditions. In the worst case, the result was equal to about 0.2 nm.

### 2.4. Experimental Setup

**Figure 1:**
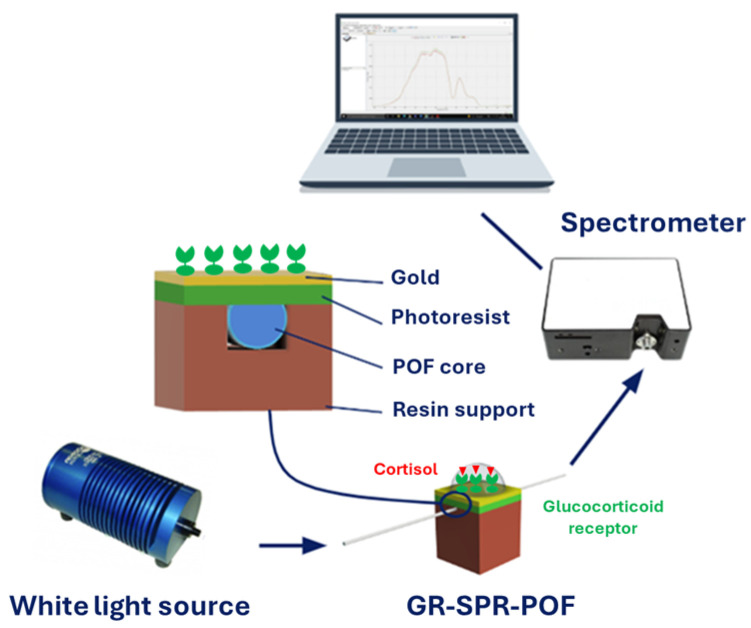
Scheme of the experimental setup employed to test the GR-SPR-POF biosensor.

The cortisol monitoring was performed via an experimental setup using a halogen lamp as a broad-spectrum light source with emission between 360 nm and 1700 nm (HL-2000LL, Ocean Insight, Orlando, FL, USA) and a spectrometer with detection range of 500−750 nm (AvaSpec-Mini2048CL-MOS2, Avantes, Apeldoorn, The Netherlands). To connect the GR-SPR−POF platform to the source and spectrometer, SMA connectors were used. The spectrometer was connected to a PC by a USB cable, allowing it to collect and process the experimental spectra. A schematization of the above-mentioned setup is shown in Figure 1.

### 2.5. Measuring Protocol

The dose–response curves of the biosensor were obtained by testing the cortisol concentrations ranging between 0.13 pM and 1100 pM in two different matrices: PBS (10 mM, pH 7.4) and artificial saliva diluted 1:50 with PBS. More specifically, the cortisol stock solution of cortisol was prepared at a concentration of 1.1 μM in water, and prior to the measurements, it was diluted with PBS or with diluted simulated saliva.

The experimental measurements were carried out by depositing 50 μL of the cortisol solution at different concentrations onto the biosensor surface for 5 min of incubation. Then, after a rinsing phase with PBS was repeated three times, the SPR spectra were acquired with PBS as a surrounding solution. All the SPR spectra were obtained using a normalization process carried out by dividing the transmitted spectra on a reference spectrum obtained in air, a medium in which the SPR condition is not fulfilled [26]. The experimental data were acquired using proprietary software (Avasoft 8, Avantes, Apeldoorn, The Netherlands) by setting an integration time of 1 ms and a number of means equal to 150. In more detail, after the normalization process was carried out using Matlab software (version R2022b, Mathworks, Natick, MA, USA), the SPR spectra were smoothed by a “smooth” Matlab function (smooth factor equal to 150) and then shifted via pure translation along the y-axis to better compare the minimum values of the SPR spectra. The resonance wavelengths were determined by windowing the functions around the resonance areas and smoothing a second time (smooth factor equal to 10); finally, a standard Matlab function was used to determine the minimum values of the spectra.

The absolute values of resonance wavelength variations ($\left|∆\lambda \right|$) at different target concentrations (c) were calculated with respect to the plasmonic spectra in the absence of cortisol as $\left|∆\lambda \right|=\left|\lambda_{c}-\lambda_{0}\right|$, and the obtained experimental data were fitted using the Langmuir model equation, as reported in Equation (1) [29].

(1) $$\left|∆\lambda \right|=\left|\Delta \lambda_{max}\right|\cdot \left(\frac{c}{K+c}\right)$$

where *λ*_c_ corresponds to the resonance wavelength when the cortisol concentration is *c*; *λ*_0_ corresponds to the resonance wavelength of the blank (solution without the cortisol); Δ*λ*_max_ is the maximum value of Δ*λ*, computed as the difference between *λ*_0_ and the one corresponding to the saturation value; and *K* is the dissociation constant.

The fitting was carried out by the Langmuir model using OriginPro software (version 9.2, Origin Lab. Corp., Northampton, MA, USA).

The analytical parameters of the GR-SPR-POF sensor in cortisol detection were calculated as the sensitivity at low concentration (*S_lowc_*), the *LOD*, and the affinity constant (*K_aff_*) using Equations (2)–(4), respectively:

(2) $$S_{lowc}=\left|\Delta \lambda_{max}\right|/K$$

(3) $$LOD=\frac{3\times {Dev.Std}_{\lambda_{0}}}{S_{lowc}}$$

(4) $$K_{aff}=\frac{1}{K}$$

## 3. Results and Discussion

**Figure 2:**
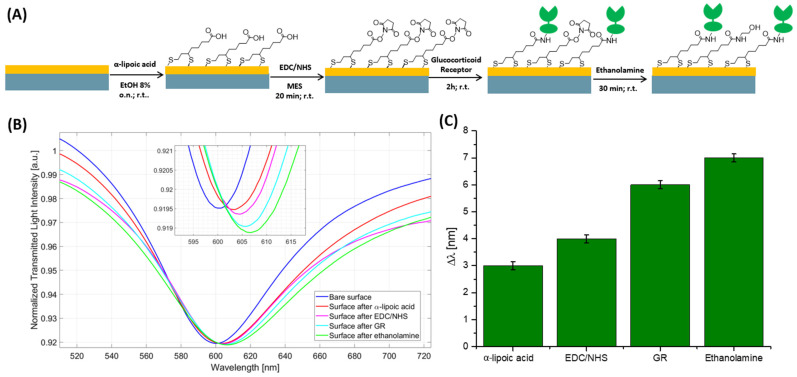
(**A**) Scheme of the functionalization protocol used. (**B**) SPR spectra attained by using PBS as bulk solution after each step of the immobilization procedure. (**C**) Variation in resonance wavelength (Δ*λ*) computed with respect to the SPR wavelength obtained on the non-functionalized chip.

The biosensor presented in this work to measure the cortisol concentration in saliva samples was obtained by coupling an SPR-POF probe [26] with the intracellular receptor GR. The functionalization of the gold surface with GR was performed according to the protocol reported in [22], along with the process described in Section 2.3 and schematically shown in Figure 2A. In the first step, the α-lipoic acid reacted with the gold surface producing the SAM; then, the activations of carboxylic groups were carried out with EDC/NHS and the activated surface was incubated with GR, which was immobilized to the SAM thanks to the formation of amidic covalent bond. Lastly, the unreacted sites were blocked with ethanolamine. The effectiveness of the coupling procedure was assessed by evaluating the plasmonic spectral changes as the resonance wavelength variation (Δ*λ*) computed in relation to that of the bare probe (i.e., without receptor) and considering the same RI surrounding solution (i.e., PBS). The SPR spectra were attained as reported in Section 2.5. As illustrated in Figure 2B, the SPR wavelength red-shifted after each phase, as depicted in the histogram in Figure 2C. This behavior is a symptom of an increase in the refractive index compared to the non-functionalized surface considering the same surrounding solution (PBS), hence confirming the success of the immobilization procedure.

**Figure 3:**
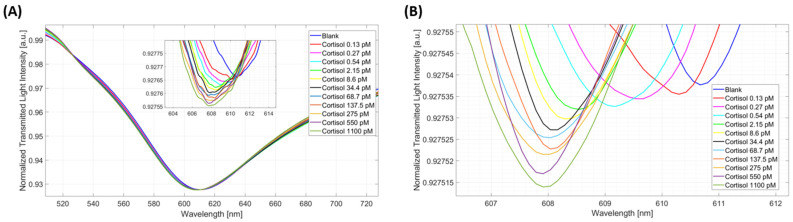
GR–cortisol binding tests in PBS. (**A**) SPR spectra, smoothed and translated along the y-axis direction, obtained in PBS at increasing cortisol concentrations. (**B**) Result of the signal processing performed to determine the resonance wavelengths useful to obtain the dose–response curve in PBS.

Then, to evaluate the biosensor’s performance in the quantification of cortisol, the GR-cortisol binding response of the biosensor was tested at different concentrations of cortisol in PBS, which ranged from 0.13 pM to 1100 pM. Figure 3A reports the SPR spectra obtained at 5 min of incubation time using the measurement protocol described in Section 2.5. In particular, the plasmonic spectra reported in Figure 3A were smoothed by the “smooth” Matlab function (smooth factor equal to 150) and shifted along the y-axis by pure translation, as described in Section 2.5.

Then, in order to obtain the dose–response curve, further signal processing was performed to determine the function minimum, i.e., the resonance wavelength. More specifically, the spectra reported in Figure 3A were windowed around the local minimum by a customized script and smoothed a second time using the “smooth” function (smooth factor equal to 10), as shown in Figure 3B. Following this processing, a standard Matlab function determined the minimum of the windowed function and, thus, the resonance wavelength at the specific concentration value.

As illustrated in Figure 3A, the resonance wavelength decreased when the cortisol concentration in PBS increased. The reported plasmonic wavelength variation achieved by the GR-cortisol binding denoted that the RI of the recognizing layer close to the gold surface decreased after binding. This trend is similar to the one attained in previous works [23,30,31].

**Figure 4:**
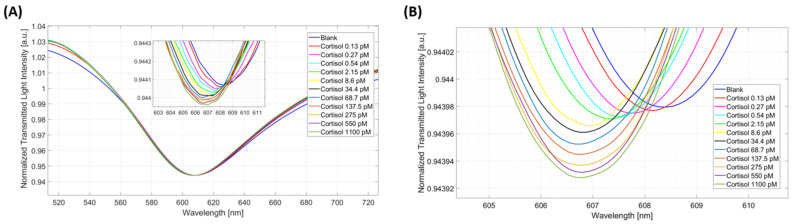
GR–cortisol binding test in artificial saliva diluted 1:50 with PBS. (**A**) SPR spectra obtained in diluted artificial saliva at increasing cortisol concentrations. (**B**) Result of the signal processing performed to determine the resonance wavelength useful to obtain the dose–response curve in artificial saliva (diluted 1:50).

Moreover, the biosensor’s response to cortisol was also tested in artificial saliva diluted 1:50 with PBS with the concentration range remaining unchanged, and the matrix effect on the biosensor’s response was evaluated. The plasmonic spectra registered in artificial saliva diluted 1:50 are shown in Figure 4A, while Figure 4B shows the results of the signal processing performed to determine the resonance wavelengths which were useful to build the dose–response curve.

**Figure 5:**
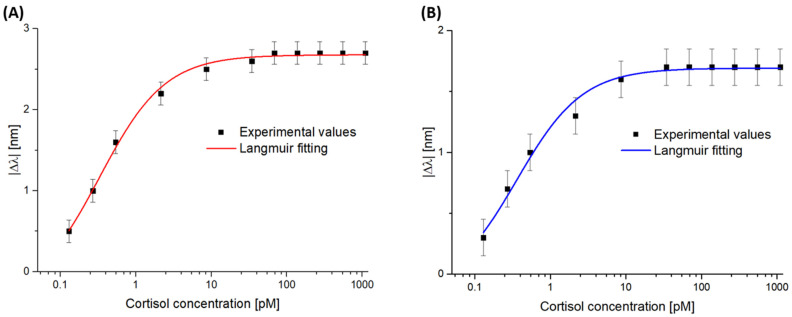
Dose–response curves obtained through cortisol monitoring in (**A**) PBS and (**B**) artificial saliva diluted 1:50. The Δ*λ* absolute values (computed in relation to the blank) as a function of increasing cortisol concentrations on the GR-SPR-POF platform are reported in a semi-log scale, along with Langmuir fitting of the experimental data.

The experimental data, meaning the absolute value of the resonance wavelength variation ($\left|∆\lambda \right|$) at varying cortisol concentrations, which was monitored in PBS and artificial saliva (diluted 1:50) and calculated with respect to the value achieved in the absence of cortisol as described above, are reported in Figure 5A,B, respectively, and fitted with the Langmuir model equation (Equation (1)) as reported in Section 2.5. The fitting parameters for both the test in PBS and that in artificial saliva (diluted 1:50) are reported in Table 1.

The parameters listed in Table 1 were used to calculate the GR-SPR-POF biosensor’s analytical parameters in relation to cortisol monitoring in both the tested matrices. Table 2 reports the results in terms of *S_lowc_*, *LOD*, and *K_aff_*. From the comparison shown in Table 2, it is possible to state that a slight matrix effect was present.

It should be stressed that the LOD value achieved in PBS by the proposed GR-SPR-POF biosensor was about one order of magnitude lower than the one (0.2 pg/mL, i.e., 552 fM) obtained in [23], where an SPR-POF probe was functionalized with an antibody receptor layer.

In fact, the choice of the GR cortisol bioreceptor used in this work was aimed at obtaining better sensor performance in terms of binding sensitivity ($S_{b}$), which can be improved by acting on both the transducer and/or the receptor, as demonstrated in [33].

Indeed, the $S_{b}$ can be calculated by the following formula [28]:

(5) $$S_{b}=(S_{1}{\times S}_{2})\times E$$

It depends on two main components: sensitivity to variations in refractive index produced by analyte binding to the receptor on the sensor surface (plasmonic sensitivity), reported as (*S*_1_ × *S*_2_), which depends on the transducer, and the efficiency (*E*) at which the presence of an analyte, at a certain concentration, is transformed into an RI variation, which depends on the characteristics of the receptor layer [33,34]. For instance, LODs in the fM range are obtained by exploiting high-efficiency receptor layers, i.e., soft molecularly imprinted nanoparticles (nanoMIP), combined with the SPR-POF probe [35].

In particular, in the present work, by maintaining an equivalent transducer (SPR-POF probe) with respect to [23], the receptor was changed to a more efficient one which converted the binding event more efficiently in RI variations thanks to its higher deformability at binding. The achievement of a lower LOD allowed for the presence of cortisol in complex matrices to be monitored by a simple dilution step, which was useful for reducing the matrix effect.

**Figure 6:**
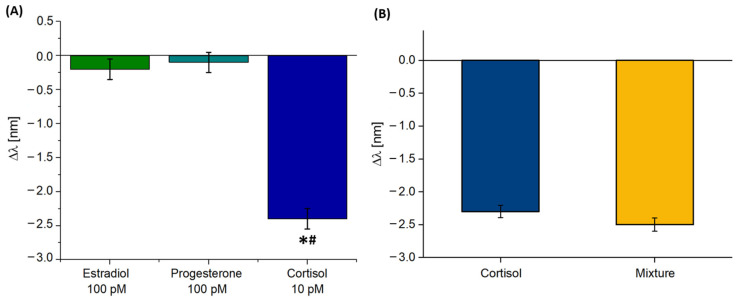
(**A**) Resonance wavelength variation for the structural analogues of cortisol (estradiol 100 pM and progesterone 100 pM) and cortisol (10 pM). One-way ANOVA * *p* < 0.01 vs. estradiol and ^#^ *p* < 0.01 vs. progesterone. (**B**) Comparison between the resonance wavelength variation achieved by a solution with cortisol only (10 pM) and with cortisol pooled in a mixture with progesterone and estradiol, each of which was considered at a concentration of 10 pM.

Moreover, the biosensor’s selectivity was tested by evaluating the biosensor’s response to hormones (i.e., estradiol and progesterone) with a molecular structure similar to cortisol. This choice was made in order to evaluate possible interferences with analytes normally registered in biological matrices with molecular structures very similar to those of cortisol, which were bio-sensitized from cholesterol as a common intermediate. The selectivity measurements were performed by examining the analogues both individually (Figure 6A) and when pooled in a mixture (Figure 6B). As shown in Figure 6A, the biosensor’s response (in terms of Δ*λ*) to estradiol and progesterone was negligible at a concentration (100 pM) one order of magnitude greater than that of cortisol (10 pM). Instead, Instead, Figure 6B shows the test carried out registering the SPR wavelength shift produced by the cortisol at 10 pM pooled in a mixture with progesterone and estradiol at the same concentration. As is clear from Figure 6B, the obtained Δ*λ* value was analogous, within the error bar, to the one produced by cortisol considered alone at 10 pM. These results confirm the good selectivity of the proposed biosensor. In addition, the glucocorticoid receptor, being an intracellular receptor, is physiologically capable of distinguishing the various target analytes in complex matrices, like a cytoplasmatic environment.

Then, to test the biosensor’s repeatability and reproducibility, several tests were carried out under similar working conditions, measuring a fixed concentration (2.15 pM) on the same regenerated platform as well as on three distinct GR-functionalized platforms after binding and regeneration cycles.

Relative standard deviations (RSD%) of 13% and 17% were obtained by testing the cortisol solution with the same regenerated biosensor three times and on three different functionalized biosensors. It should be stressed that the RSD% value relative to the repeatability was higher than 10% because of a slight worsening in the sensor response after subsequent regeneration cycles, which was probably due to a partial deterioration of the recognition layer. Concerning the reproducibility, a RSD% higher than 10% could be attributable to the non-industrial scale of biosensor production, which can lead to a certain variability caused by a changeability in both SPR platform fabrication and the functionalization process.

The regeneration procedure consisted of a well-established protocol based on a glycine/HCl solution (10 mM, pH 2.2) treatment step and successive storage at 4 °C in PBS [22]. More specifically, it consisted of two washing steps with 50 μL of glycine/HCl solution involving 5 min of incubation time at each step. The regeneration was evaluated as efficient within three regeneration cycles over one month. The results achieved by these tests provide a good confirmation of the repeatability, reproducibility, and reusability of the proposed sensor within the mentioned time period. Despite this, a gradual worsening of the response was obtained outside of this time-frame.

Finally, the sensor performances, in terms of LOD and working range, were assessed with respect to other SPR-based sensors reported in the literature for cortisol detection in similar matrices. As reported in Table 3, is possible to state that, to the best of our knowledge, the proposed cortisol sensor showed the best performance in terms of LOD and a working range of over three orders of magnitude.

As it is possible to deduce from Table 3, the GR-based recognition layer improved the biosensor performances with respect to the antibody-based one.

The improvement of SPR biosensors in target monitoring, when passing from an antibody-based recognizing layer to a bioreceptor-based one, was confirmed by Arcadio et al. [22], who focused on estradiol detection via a highly efficient bioreceptor (Estrogen Receptor, ER) utilized as a recognition element for estradiol instead of a specific antibody. In [22], in terms of LOD, the obtained biosensor performance improved by about two orders of magnitude with respect to another SPR immunoassay functionalized with an anti-estradiol antibody [39]. This improvement was attributable to the drastic conformational changes in the bioreceptor at binding, as reported in [40]. An analogous behavior can be associated with the GR-based bioreceptor layer. Indeed, GR, belonging to the same family of intracellular hormone receptors, produces important conformational changes at binding with its target, as reported in [28].

Table 4 summarizes the improvement achieved by using bioreceptors instead of antibody layers for two different analytes, i.e., estradiol and cortisol.

## 4. Conclusions

In this study, the fabrication and characterization of a GR-SPR-POF biosensor for the monitoring and quantification of cortisol in saliva was described.

The plasmonic POF-based biosensor was obtained by functionalizing the plasmonic POF platform with the GR receptor layer, a highly efficient bioreceptor, as the cortisol recognition element.

The proposed biosensor was successfully tested in PBS and simulated saliva. The biosensor response showed an LOD of 59 fM, allowed for rapid detection (5 min), and enabled the determination of the cortisol concentration in artificial saliva with a working range of 0.13–36 pM, thus encompassing the expected load for samples diluted 1:50 in real scenarios without any extraction process or sample pretreatment.

In particular, the proposed sensor system could be employed as a POCT to allow for easy periodical screening of cortisol in saliva in several application fields.

## Figures and Tables

**Table 1:** Langmuir fitting parameters obtained for cortisol monitoring in PBS and diluted artificial saliva.

Matrix	*λ*_0_ [nm]	Δ*λ*_max_ [nm]	K [pM]	Statistics	
				χ^2^	R^2^
PBS	−0.35 ± 0.16	2.67 ± 0.02	0.33 ± 0.04	0.067	0.995
Artificial saliva (diluted 1:50)	−0.15 ± 0.15	1.69 ± 0.02	0.36 ± 0.08	0.140	0.987

**Table 2:** Analytical parameters of the sensor obtained for cortisol detection in PBS and diluted artificial saliva.

Matrix	*S_lowc_* (|Δ*λ_max_*|/*K*)	*LOD*$\;({(3\times Dev.Std}_{\lambda_{0}})$/*S_lowc_*) [32]	*K_aff_* (1/*K*)
PBS	8.09 nm/pM	59 fM	3.03 pM^−1^
Artificial saliva (diluted 1:50)	4.69 nm/pM	96 fM	2.78 pM^−1^

**Table 3:** Comparison between SPR cortisol biosensors in terms of LOD and working range.

Sensor Configuration	Matrix	Working Range	LOD	Ref.
D-shaped silica optical fiber coupled with antibody	PBS	0.01–100 ng/mL	1.46 ng/mL	[24]
AuPd unclad POF coupled with antibody	PBS	0.005−10 ng/mL	1 pg/mL	[25]
Commercial chip coupled with antibody	PBS	1.5–10 ng/mL	0.36 ng/mL	[36]
Commercial chip coupled with antibody	Saliva	1.5 ng/mL	1 ng/mL	[36]
Commercial chip coupled with antibody	Saliva	9–132 ng/mL	4 ng/mL	[37]
Commercial chip coupled with antibody	Saliva	91–934 pg/mL	49 pg/mL	[38]
SPR-POF chip coupled with antibody	PBS	0.1–10 pg/mL	0.2 pg/mL	[23]
SPR-POF chip coupled with antibody	Seawater	0.1–10 pg/mL	0.8 pg/mL	[23]
SPR-POF chip coupled with GR	PBS	0.13–36 pM (0.047–13 pg/mL)	59 fM (0.021 pg/mL)	This work
SPR-POF chip coupled with GR	Artificial saliva	0.13–36 pM (0.047–13 pg/mL),	96 fM (0.034 pg/mL)	This work

**Table 4:** Comparison between SPR biosensors based on antibody and bioreceptor layers for two different analytes.

Transducer	Receptor	Analyte	LOD	Ref.
Commercial chip	Antibody	Estradiol	1 pg/mL	[39]
Spoon-shaped waveguides	ER	Estradiol	0.027 pg/mL	[22]
AuPd-coated POF	Antibody	Cortisol	1 pg/mL	[25]
SPR-POF	GR	Cortisol	0.021 pg/mL	This work

## Data Availability

The data are available upon reasonable request from the corresponding author.
